# Long-acting insulin analogues for type 1 diabetes: An overview of systematic reviews and meta-analysis of randomized controlled trials

**DOI:** 10.1371/journal.pone.0194801

**Published:** 2018-04-12

**Authors:** Fernanda O. Laranjeira, Keitty R. C. de Andrade, Ana C. M. G. Figueiredo, Everton N. Silva, Mauricio G. Pereira

**Affiliations:** 1 Faculty of Health Sciences, University of Brasilia, Brasilia, Federal District, Brazil; 2 Faculty of Medical Sciences, University of Brasilia, Brasilia, Federal District, Brazil; Weill Cornell Medical College Qatar, QATAR

## Abstract

**Background:**

The comparison between long acting insulin analogues (LAIA) and human insulin (NPH) has been investigated for decades, with many randomized controlled trials (RCTs) and systematic reviews giving mixed results. This overlapping and contradictory evidence has increased uncertainty on coverage decisions at health systems level.

**Aim:**

To conduct an overview of systematic reviews and update existing reviews, preparing new meta-analysis to determine whether LAIA are effective for T1D patients compared to NPH.

**Methods:**

We identified systematic reviews of RCTs that evaluated the efficacy of LAIA glargine or detemir, compared to NPH insulin for T1D, assessing glycated hemoglobin (A1C) and hypoglycemia. Data sources included Pubmed, Cochrane Library, EMBASE and hand-searching. The methodological quality of studies was independently assessed by two reviewers, using AMSTAR and Jadad scale. We found 11 eligible systematic reviews that contained a total of 25 relevant clinical trials. Two reviewers independently abstracted data.

**Results:**

We found evidence that LAIA are efficacious compared to NPH, with estimates showing a reduction in nocturnal hypoglycemia episodes (RR 0.66; 95% CI 0.57; 0.76) and A1C (95% CI 0.23; 0.12). No significance was found related to severe hypoglycemia (RR 0.94; 95% CI 0.71; 1.24).

**Conclusion:**

This study design has allowed us to carry out the most comprehensive assessment of RCTs on this subject, filling a gap in diabetes research. Our paper addresses a question that is important not only for decision makers but also for clinicians.

## Introduction

Intensive insulin therapy improves outcomes of patients with type 1 diabetes (T1D). These improvements include better glycemic control and a reduction in the risk of complications [1–3], preventing or delaying the progression of chronic microvascular complications in approximately 50% of cases [1], which makes the treatment more effective. Although the importance of glycemic control has been known for approximately three decades [4], this control is still flawed in many countries. For example, glycemic control was unsatisfactory in 87% of Brazilian T1D patients (glycated hemoglobin, A1C > 7%) in 2012 [5]. This scenario has become a global concern since the number of people living with diabetes has almost quadrupled since 1980 to 422 million adults (6). It is estimated that T1D accounts for between 5 and 10% of all cases of diabetes (6), and the incidence rate increases by 3% a year [6].

The comparison between insulin analogues and human insulin has been investigated for decades, with many randomized controlled trials (RCTs) and systematic reviews giving mixed results [7–11] This contradictory evidence has increased uncertainty on coverage decisions at health system level. For example, among the five countries with the highest number of people with T1D (China, India, the USA, Brazil and Russia), only the USA [12] and Russia [13] have clinical guidelines for T1D patients that include long-acting insulin analogues among the treatment options, under restricted indications.

To deal with the substantial increase in the number of overlapping systematic reviews, guidance on how to conduct overviews of systematic reviews has emerged since the mid-2000s [14,15]. The purpose of overviews is to summarize evidence, synthesizing results from multiple systematic reviews into a single, useful document [15,16]. Overviews identify high-quality, reliable systematic reviews and explore consistency of findings across reviews [15]. To the best of our knowledge, this is the first overview on the efficacy of long-acting insulin analogues compared to NPH for T1D. We sought to shed some light on this issue to support decision making from both clinical and public health perspectives.

In this context of doubt and uncertainty on the recommendations [17,18] we aimed to conduct an overview of systematic reviews and also update existing reviews, aggregating clinical trials published to date and preparing a new meta-analysis to determine whether long-acting insulin analogues are effective and safe for T1D patients compared to NPH in reduction of hypoglycemia and maintenance or improvement of glycemic control.

## Materials and methods

We performed an overview of systematic reviews of RCTs (guided by the Cochrane Handbook [15]) on the efficacy of long-acting insulin analogues compared to NPH human insulin for T1D patients. We also updated systematic reviews included in this overview, by including RCTs published after publication of the systematic reviews. The protocol for this overview was registered in the International Prospective Register of Systematic Reviews (PROSPERO) under number CRD42016047137. We report according to PRISMA guidelines (S1 Checklist).

### Criteria for study selection

We selected systematic reviews of RCTs that evaluated efficacy of long-acting insulin analogues compared to NPH human insulin for T1D patients. The inclusion criteria were: i) a direct or indirect comparison between long-acting insulin analogues and NPH; ii) T1D patients regardless age group; iii) results that included at least one of the primary outcomes related to efficacy (A1C, and general, severe and nocturnal hypoglycemia).

In cases of eligible systematic reviews that included RCTs and other study designs, we included these reviews if it was possible to extract the RCTs results independently. A similar procedure was applied for reviews that reported results for type 1 and type 2 diabetes. If several publications from the same author or group were identified, the publications were re-scanned to decide whether the reported reviews or trials were the same. In such cases, the most recent publication was selected unless the earlier one provided more information. Studies published as abstracts were included when sufficient information on methods and results was provided. We restricted our search to English, Spanish and Portuguese systematic reviews and studies in humans. There was no restriction on year of publication. We included both Cochrane and non-Cochrane reviews. We excluded studies that assessed pregnant patients, analyzed only rapid-acting insulin analogues as the intervention, or assessed outcomes other than A1C and hypoglycemia.

To update the included systematic reviews, we performed a separate search for RCTs assessing long-acting insulin analogues as the intervention compared to NPH human insulin, and reporting outcomes as A1C (in terms of the difference between the end of study and baseline or the measures of the baseline and the end of study independently) and hypoglycemia, considering the categories: general, severe and nocturnal (in terms of number of episodes or rate of episodes per person-time). Regarding the clinical trials located in the selected systematic reviews or in the complementary search, we considered only phase III RCTs, classified as Jadad ≥ 2, which compared long-acting insulin analogues to NPH, evaluating the outcomes of interest for inclusion in meta-analysis.

### Data sources and searches

Potentially relevant systematic reviews were identified through a comprehensive and exhaustive search of electronic databases: the Cochrane Library, PubMed and EMBASE. We conducted the first search in February 2015 and the last update in October 2016. We used a filter for systematic reviews and meta-analyses and for study language when this option was available at the database searched.

The search strategy was developed using MeSH terms for Pubmed, EMTREE terms for Embase, and a combination of keywords for Cochrane Library. For example, the full electronic search strategy used at Pubmed was: ("Diabetes Mellitus, Type 1"[Mesh] OR "Type 1 Diabetes Mellitus"[tiab] OR "IDDM"[tiab] OR "T1DM"[tiab]) AND (("long-acting insulin analogue"[tiab] OR (("Analog Sci Fict Sci Fact"[Journal] OR "analog"[All Fields]) AND ("insulin, long-acting"[MeSH Terms] OR ("insulin"[All Fields] AND "long-acting"[All Fields]) OR "long-acting insulin"[All Fields] OR ("long"[All Fields] AND "acting"[All Fields] AND "insulin"[All Fields]) OR "long acting insulin"[All Fields])) OR "long-acting analog* insulin"[All Fields] OR "basal insulin analogue"[tiab] OR "basal analog* insulin*"[All Fields] OR "insulin* analog*"[All Fields]) OR ("glargine"[tiab] OR "lantus"[tiab] OR "HOE 901"[tiab]) OR ("insulin detemir"[Supplementary Concept] OR "detemir"[tiab] OR "levemir"[tiab] OR "NN304"[tiab]) OR ("insulin degludec"[Supplementary Concept] OR "insulin degludec"[tiab] OR "degludec "[tiab])) AND (Meta-Analysis[ptyp] OR systematic[sb]) AND (English[lang] OR Portuguese[lang] OR Spanish[lang]). This strategy was slightly adapted for use in EMBASE and Cochrane Library (S1 Table).

We also performed a separate search to check for RCTs published after the most recent systematic review included in our overview. We used the following strategy in Pubmed (from 09/01/2013 to 29/10/2016): ("Diabetes Mellitus, Type 1"[MeSH] OR "Type 1 Diabetes Mellitus"[tiab] OR "IDDM"[tiab] OR "T1DM"[tiab]) AND (("long-acting insulin analogue"[tiab] OR ("analog"[All Fields]) AND ("insulin, long-acting"[MeSH Terms] OR ("insulin"[All Fields] AND "long-acting"[All Fields]) OR "long-acting insulin"[All Fields] OR ("long"[All Fields] AND "acting"[All Fields] AND "insulin"[All Fields]) OR "long acting insulin"[All Fields])) OR "long-acting analog* insulin"[All Fields] OR "basal insulin analogue"[tiab] OR "basal analog* insulin*"[All Fields] OR "insulin* analog*"[All Fields]) OR ("glargine"[All Fields] AND (Supplementary[All Fields] AND Concept[All Fields]) OR "glargine"[tiab] OR "lantus"[tiab] OR "HOE 901"[tiab]) OR ("insulin detemir"[All Fields] AND (Supplementary[All Fields] AND Concept[All Fields]) OR "detemir"[tiab] OR "levemir"[tiab] OR "NN304"[tiab]) OR ("insulin degludec"[All Fields] AND (Supplementary[All Fields] AND Concept[All Fields]) OR "insulin degludec"[tiab] OR "degludec"[tiab] OR "tresiba"[tiab])) AND (English[lang] OR Portuguese[lang] OR Spanish[lang]) AND (Randomized Controlled Trial[ptyp] OR Clinical Trial, Phase III[ptyp]).

The literature search was supplemented by a hand search for abstracts at specialized scientific journals on diabetes, conferences and meetings websites. We also search for unpublished studies at clinicaltrials.gov. In cases of incomplete data, authors were contacted to obtain additional information.

### Data collection and analysis

Titles and abstracts were screened by two independent investigators (FOL and KRCA). Duplicates and those that did not meet the eligibility criteria were excluded. The remaining records were read in their entirety, and those suitable for the overview were selected. Disagreements were solved by consensus.

### Data extraction and quality assessment

Two researchers (FOL and KRCA) independently extracted data on to a standardized datasheet. In cases of disagreement, decisions were made by consensus. From systematic reviews, full reference, authors, year of publication, and included clinical trials were collected. For clinical trials found in the selected systematic reviews and the complementary search, we collected full reference, authors, year of publication, characteristics of participants, number of individuals in the study and in each comparison arm, description of interventions in each comparison group (including brand of insulin, frequency per day, period of day, associated bolus insulin), and information on the selected outcomes. For continuous variables (A1C), we collected the mean difference (SMD) between the end of the study and the baseline and the standard deviation (SD) for all comparison groups. In case of missing data (i.e.SD), we tried to contact the authors, and in cases of no reply, we assumed SD = 0.05. For dichotomous variables (general, severe and nocturnal hypoglycemia), we used the number of episodes per person-week, which is calculated by dividing the total number of episodes in each group by the number of persons in each group adjusted by the time until a hypoglycemia occurs. When studies reported only the rate of episodes per person-time (time other than a week), we disaggregated this rate to obtain the number of episodes. For cross-over trials, we considered the A1C mean difference in the first period and the sum of hypoglycemia episodes in both periods of analysis. For trials that analyzed two intervention groups (i.e.: the same drug but different frequency or time of injection), we considered the best A1C results and summed the hypoglycemia episodes of both groups. This was different from Rossetti and colleagues [19], who yielded the hypoglycemia results as episodes per person-time, thus we considered the best results, as it was impossible to sum the rates.

In the case of two or more publications from the same study, we use the most complete in the analysis. In the case of trials reported in conference abstracts and published later, we refer to the full text.

Quality of systematic reviews was assessed using the AMSTAR tool [20]. Quality of the evidence for RCTs was assessed using Jadad modified scale [21]. The original Jadad scale is formed by seven questions about the methodological quality of clinical trials. The modified Jadad scale has five questions, specifically: Was the study described as randomized? Was the method of randomization appropriate? Was the study described as double blind? Was the method of blinding appropriate? Was there a description of withdrawals and dropouts? Clinical trials with Jadad score less than 2 were excluded.

### Data synthesis and analysis

A random effects meta-analysis was chosen a priori. For continuous data (A1C) we used the difference between the end of the study and the baseline and the SD for all comparison groups to calculate the mean difference as associate effect measure, and 95% confidence intervals by DerSimonian & Laird method. For dichotomous data (general, severe and nocturnal hypoglycemia) we calculated the relative risk as the effect measure. The measure for any type of hypoglycemia considered in the analysis was number of episodes per person-week, so the relative risk should be interpreted as incidence density, likewise 95% confidence intervals by the Mantel-Haenszel method. For dichotomous outcomes in which studies reported 0 events in one treatment arm, we added 0.5 to the numerator and 1 to the denominator.

It is worth noting that for dichotomous outcomes the N in forest plots does not represent the number of individuals for each group in each study, but the number of person-week.

The chi-squared test was applied to measure heterogeneity between studies at the p < 0.10 significance level. We adopted this p-value over the traditional p < 0.05 to be more conservative as low power is attributed to the chi-squared test in meta-analyses when a small number of studies or studies of small sample size are considered [22]. The magnitude of inconsistency was measured using I-squared (I^2^) statistics. An I^2^ of 80% was considered high heterogeneity. For results above this threshold, we conducted sensitivity analyses to determine whether results of some subgroups separately affected the results. We performed subgroup analyses for (a) age group (adult and pediatric); (b) type of bolus insulin (i.e., regular human insulin, lispro, aspart or both); and (c) brand of long-acting insulin analogue assessed (i.e., glargine or detemir).

When heterogeneity could not be explained by subgroup analysis, we performed a meta-regression analysis, considering the variables: (a) age group; (b) type of bolus insulin; (c) brand of long-acting insulin analogues assessed; (d) Jadad score; and (e) frequency per day of insulin analogues; (f) if study design was cross-over or not; (g) follow-up period; (h) definition of hypoglycemia (just for general hypoglycemia); and if study had provided standard deviation for A1C measures.

We assessed the potential for publication bias in meta-analyses using funnel plots and Egger’s test [23–25].

We used Stata version 11.0 for the statistical analysis.

## Results

### Description of included reviews

**Fig 1:**
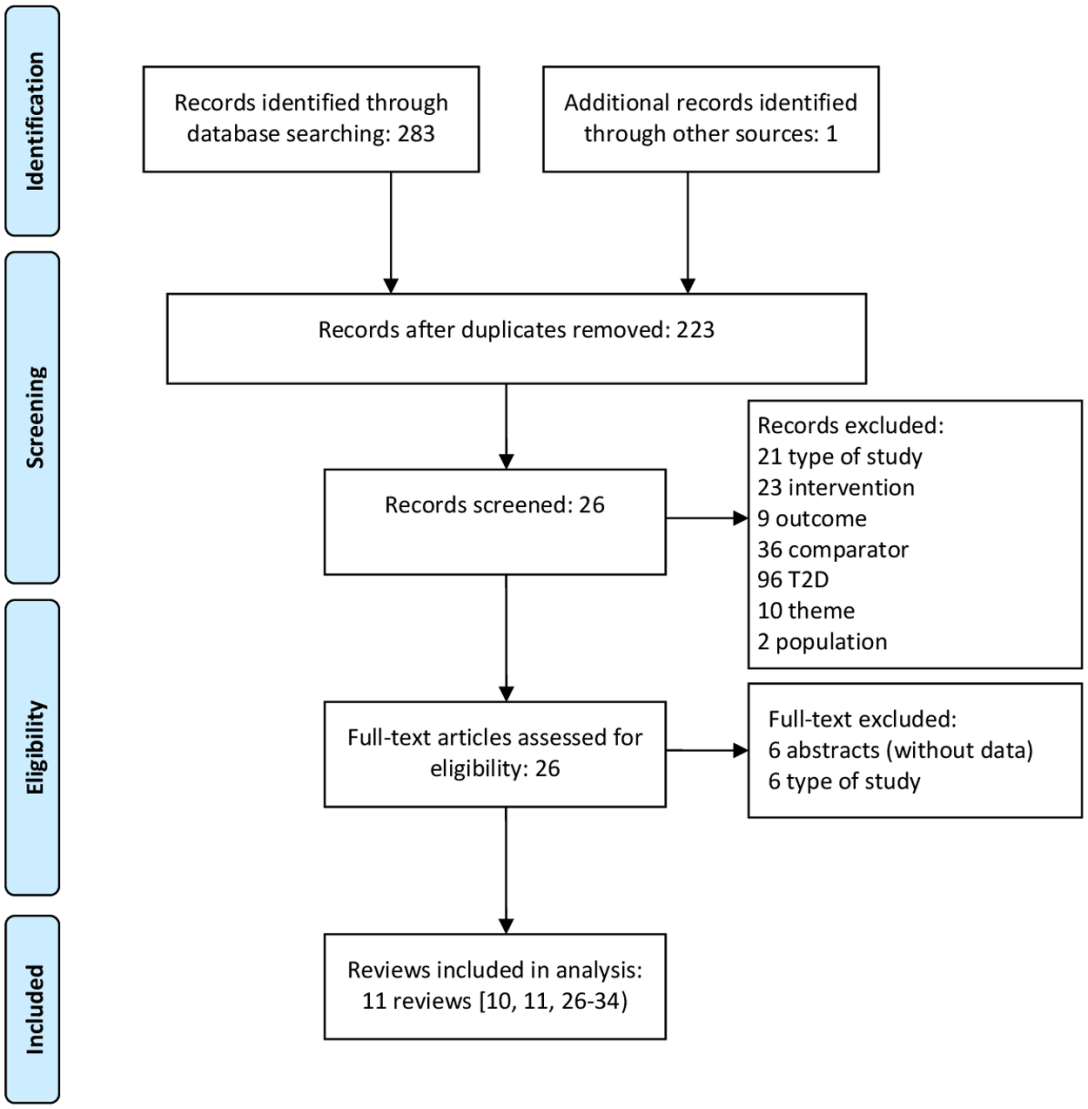
Flowchart of the study selection process for systematic reviews.

Our search for systematic reviews yielded 284 references, of which 61 were duplicates. After removing duplicates and assessing titles and abstracts, 26 studies were identified for full-text reading. References excluded in this first phase were those outside the inclusion criteria. After the screening of full texts, 11 systematic reviews [10,11,26–34] (three had been published twice [35–37]) met the overview inclusion criteria (Fig 1). Among the 11 included studies, we found seven original systematic reviews, four with meta-analysis [28,30–32] and three without meta-analysis [26,33,34], two indirect comparisons [10,11] and two complete health technology assessment studies [27,28]. Characteristics of included studies from the eligibility phase are described in S2 Table.

Altogether, the included systematic reviews yielded 62 references of RCTs. Among them, we included 25 in the analysis and excluded 37 that did not meet the pre-defined inclusion criteria or that did not measure selected outcomes appropriately. Characteristics of included trials are available in S3 Table. The distribution of selected clinical trials among included in the systematic reviews is presented in Table 1.

**Table 1:** Distribution of considered clinical trials among included systematic reviews.

Trials / Systematic reviews	Wang et al, 2003 [26]	Warren et al, 2004 [27]	Mullins et al, 2007 [28]	Tran et al, 2007[29]	Vardi et al, 2008 [30]	Singh et al, 2009 [31]	Sanches et al, 2011[11]	Szypowska et al, 2011 [32]	Frier et al, 2013[33]	Souza et al, 2014 [34]	Tricco et al, 2014 [10]
Raskin et al, 2000 [38]	**x**	**x**	**x**	**x**	**x**	**x**	**x**			**x**	**x**
Ratner et al, 2000 [39]	**x**	**x**	**x**	**x**	**x**	**x**	**x**				**x**
Hermansen et al, 2001 [40]	**x**		**x**	**x**	**x**						
Schober et al, 2002 [41]	**x**		**x**	**x**	**x**	**x**				**x**	
Rossetti et al, 2003 [42]			**x**	**x**	**x**	**x**					**x**
Vague et al, 2003 [43]				**x**	**x**	**x**	**x**	**x**	**x**		**x**
Murphy et al, 2003 [44]					**x**	**x**					
Porcellati et al, 2004 [45]				**x**	**x**	**x**	**x**				**x**
Hermansen et al, 2004 [46]					**x**	**x**	**x**		**x**		
Home et al, 2004 [47]				**x**	**x**	**x**	**x**	**x**	**x**		**x**
Russel-Jones et al, 2004 [48,49]				**x**	**x**	**x**	**x**	**x**	**x**		**x**
Standl et al, 2004 [50]				**x**		**x**			**x**		**x**
Home et al, 2005 [51]				**x**	**x**	**x**	**x**				
Fulcher et al, 2005 [52]				**x**	**x**	**x**	**x**				**x**
Pieber et al, 2005 [53]				**x**		**x**	**x**	**x**	**x**		**x**
NN 304–1476 [54]								**x**			
Ashwell et al, 2006 [55]					**x**	**x**					**x**
Kolendorf et al, 2006 [56]				**x**	**x**	**x**		**x**	**x**		**x**
Robertson et al, 2007 [57]				**x**	**x**	**x**		**x**			
Chatterjee et al, 2007 [58]					**x**	**x**				**x**	**x**
Mianovska et al, 2007 [59]						**x**					
Chase et al, 2008 [60]						**x**				**x**	
Bartley et al, 2008 [61]							**x**	**x**	**x**		**x**
Hassan et al, 2008 [62]											
Bolli et al, 2009 [63]						**x**	**x**				**x**

In Table 1 we can observe that none of the included systematic reviews analyzed all the available clinical trials selected by this overview. Concerning completeness, the most complete included systematic review was Singh et al. [31], with 21 clinical trials. This does not mean that the reviews did not include more trials than analyzed here, but that some previously assessed trials were not included in this overview analysis, due to the inclusion criteria.

The complementary search for clinical trials yielded 49 references just from Pubmed. After screening of titles, abstracts and full texts, we included three references [64–66]. Twenty-one references were excluded due to inappropriate comparison (to other comparator), 11 due to inappropriate interventions, four of each due to unsuitable outcome and study type, and two of each due to unsuitable population, diverse theme, and duplicates.

### Methodological quality of included studies

Mean methodological quality of the 11 included systematic reviews was 7.28 (SD 3.03). Six of them presented methodological quality ranging from 8 to 11 points out of a maximum of 11 points on the AMSTAR score (S4 Table). Almost all studies conducted a comprehensive literature search, evaluated scientific quality of the primary studies and used an appropriate method to combine the results. However, only three studies assessed the likelihood of publication bias [10,30,31], and only four provided lists of both included and excluded studies [27–30,32]. No systematic review was excluded because of poor methodological quality. We considered that the main function of systematic reviews in this overview was to attend as a source for published clinical trials.

### Quality of evidence in included reviews

Combining the clinical trials located in the systematic reviews included in this overview and those found in the complementary search, 28 randomized clinical trials were analyzed. It is worth noting that the overview protocol has already indicated our decision to accept the fact that it was impossible to blind patients in these studies because the compared insulins have a different appearance; long-acting insulin analogues are transparent and NPH insulin is a suspension. Even if the authors had chosen individual pre-prepared doses, study subjects could trigger the injection mechanism (syringe or pen) and observe the color of the medication. In addition, a double-dummy strategy would not be recommended because it is considered unethical in this case. We therefore decided to include studies with Jadad ≥ 2, since the lack of blindness necessarily takes 2/5 points off the score. However, after analyzing the quality of all the studies, it was clear that evaluators’ blindness was possible, and was successfully performed in three of the included studies.

Concerning the methodological quality of evidence from these studies, they were all randomized and reported data for all individuals (per protocol, by intention-to-treat analysis, or both), justifying withdrawals and dropouts. Half the clinical trials presented appropriate randomization methods and just three had any level of blindness (S1 Fig).

### Quantitative analysis

#### Meta-analysis

Among all 28 included clinical trials, allocating 8158 individuals (cross-over studies counting twice), 4601 were randomized for insulin analogues and 3557 for NPH insulin. Seven references were cross-over studies [40,44,55,56,58,59,66] and four were non-inferiority trials [54,61,64,65]. Concerning quantitative analysis, the outcomes that were most frequently analyzed were A1C and general hypoglycemia (25 trials).

**Fig 2:**
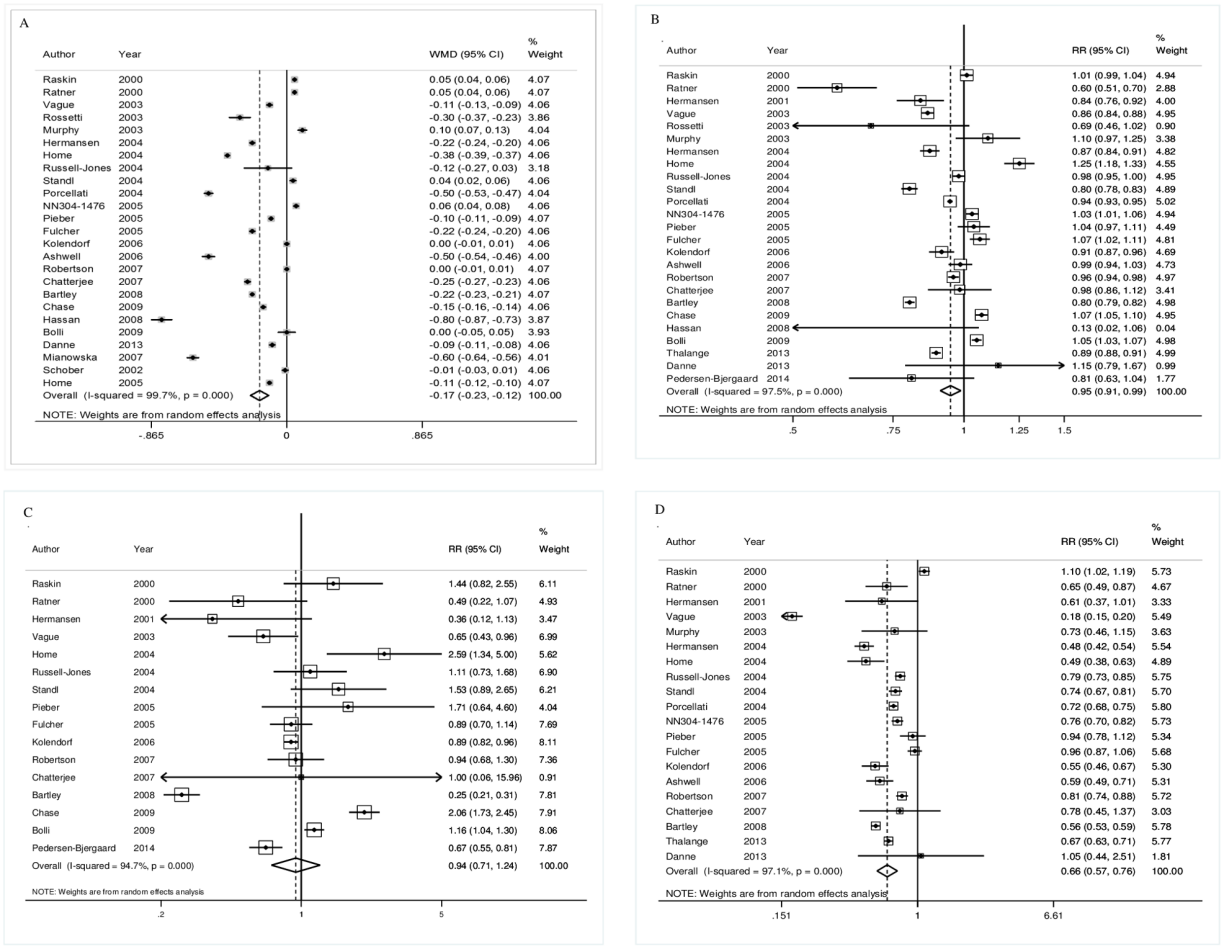
Meta-analysis of outcomes assessed in this overview: A: glycated hemoglobin—A1C; B: general hypoglycemia; C: severe hypoglycemia; D: nocturnal hypoglycemia.

In relation to A1C, the long-acting insulin analogue yielded a statistically significant reduction in A1C mean difference, considering the difference between end of study and baseline (95% CI -0.23; -0.12; I^2^ 99.7%) compared to NPH insulin (Fig 2A). This difference is clinically significant, as a difference in A1C of 1.0% is already considered to be a minimal clinical threshold [67].

Regarding hypoglycemia, its definition varied among the analyzed trials. Furthermore, authors have classified hypoglycemia into many categories, such as general, overall, minor, severe, major, symptomatic, all-day, day and nocturnal. We tried to group these categories into understandable classes; for instance, we included general, overall, minor and all day in the ‘general’ class, severe and major in the ‘severe’ class and maintained nocturnal as a separate class. The category “symptomatic” was assigned to the general or severe class, depending on the applied definition in the studies.

Considering general hypoglycemia, the meta-analysis showed a statistically significant difference between the insulin analogue and NPH (RR = 0.95; 95% CI 0.91; 0.99; I^2^ 97.5%) (Fig 2B). Although the effect was statistically significant, its magnitude and clinical significance were small.

Data for severe hypoglycemia were usefully available from 16 studies (Fig 2C). Concerning this outcome, the pooled estimate of all trials using the random effects model showed long-acting insulin analogues did not have statistically significant results compared to NPH, revealing a RR = 0.94 (95% CI 0.71; 1.24; I^2^ 94.7%).

Concerning nocturnal hypoglycemia, it was available from 20 clinical trials, and the meta-analysis results favor insulin analogues (RR 0.66; 95% CI 0.57; 0.76; I2 97.1%), meaning that the risk of having a hypo episode was reduced (Fig 2D).

#### Meta-regression

Heterogeneity was impressive for all analyzed outcomes, being above 90%. Concerning A1C, the high heterogeneity was partially explained in 15.96% by methodological quality (p = 0.07) and in 9.16% by duration of the study, but not statistically significant (p = 0.16). The heterogeneity in general hypoglycemia was explained in 26.13% by bolus insulin (p = 0.01), in 13.96% by hypoglycemia definition (p = 0.10), and in 7.16% by duration of study (p = 0.10). For nocturnal hypoglycemia, it was explained in 23.71% by crossover design (p = 0.08) and in 13.55% by intervention brand (p = 0.08). For severe hypoglycemia, heterogeneity was marginally explained by age in 3.84% (p = 0.29).

#### Analysis of publication bias

**Fig 3:**
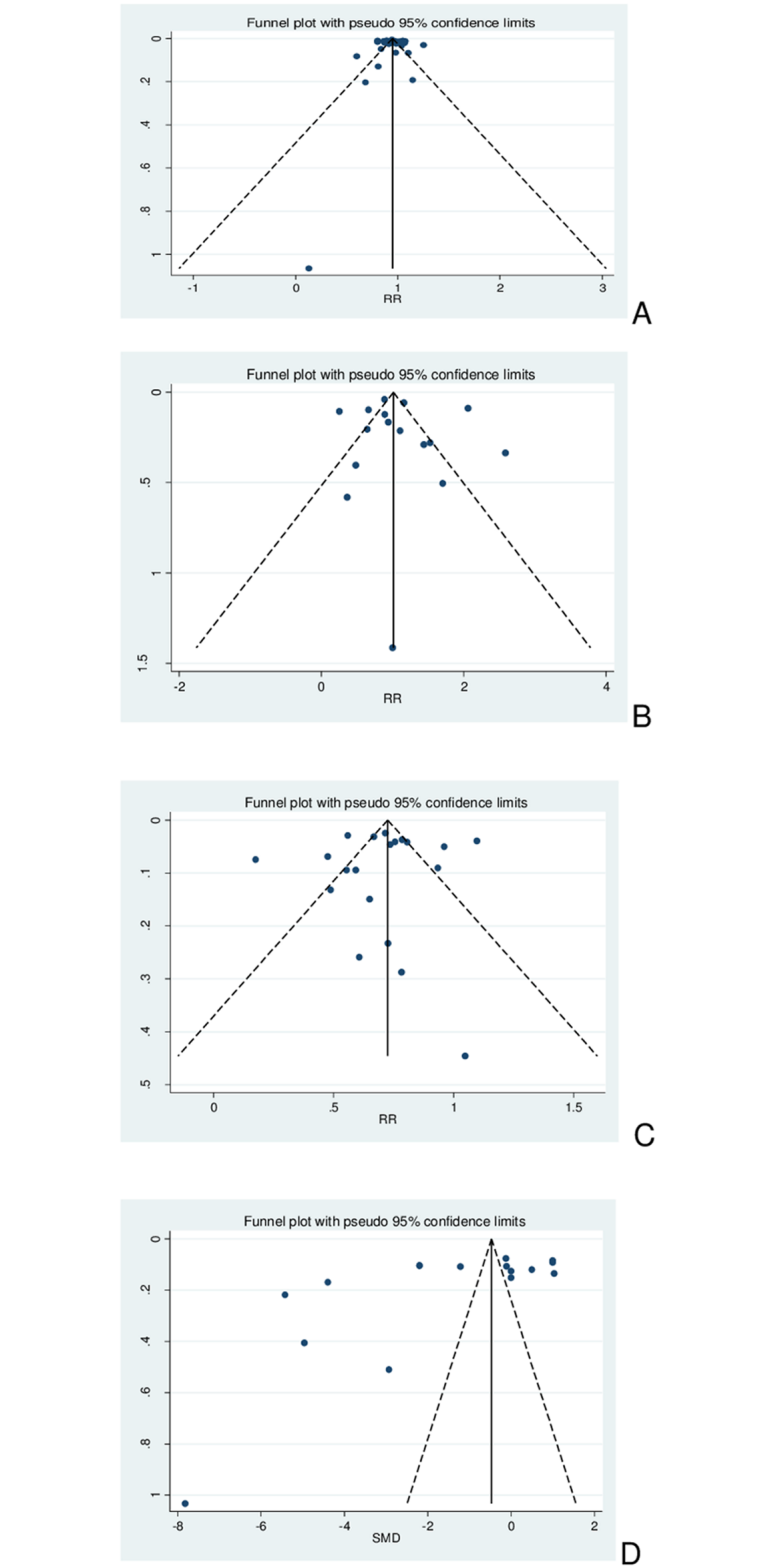
Publication bias analysis by Begg’s funnel plots. A. General hypoglycemia. B. Severe hypoglycemia. C. Nocturnal hypoglycemia. D A1C.

Visual inspection of Begg’s funnel plots for hypoglycemia endpoints (Fig 3A, 3B and 3C) showed a tendency of published trial results to have a small risk of publication bias and the Egger’s test confirmed this direction for general (p = 0.75), severe (p = 0.68), and nocturnal hypoglycemia (p = 0.74). However, A1C studies were very dispersed (Fig 3D), suggesting that there is publication bias concerning this outcome, and this was confirmed by Egger’s test (p = 0.05).

## Discussion

To the best of our knowledge, this study is the first overview of systematic reviews to assess the efficacy of long-acting insulin compared to NPH. This study design has allowed us to carry out the most comprehensive assessment of RCTs on this subject, filling a gap in diabetes research, since there are many overlapping systematic reviews with contradictory recommendations. We found evidence that long-acting insulin analogues are efficacious compared to NPH, with estimates showing a reduction in nocturnal hypoglycemia episodes (RR 0.66) and general hypoglycemia episodes (RR = 0.95). Further results showed a non-clinically significance reduction in A1C.

Our study used appropriate outcome measures for the outcomes included in the analysis, like other previously published systematic reviews [28,30]. Of the 11 reviews included in this overview, only three [28,30,31] reported the measure of hypoglycemia as episodes per person-time, as recommended by methodological guidelines [15,68]. The other reviews [10,11,26,29,32–34] reported hypoglycemia as the risk of having at least one event during the study (number of people who had hypoglycemia / number of people in the study group). This is a misconception, given that each person could experience more than one event during the whole study follow-up. Regarding the A1C measure, three reviews [31,33,34] reported only the comparison of the final A1C between groups. Using such a measure is not appropriate, since the measure of interest should be how much the interventions have decreased A1C, it being more appropriate to measure A1C difference between the end of the study and baseline [69], as we reported.

Among the systematic reviews that assessed outcomes similar way to this overview, Vardi and collaborators [30], in a Cochrane review, yielded comparable results to our overview concerning hypoglycemia. Results showed the reduction was not statistically significant in general hypoglycemia (OR 0.93; 95% CI 0.8; 1.08) (30). For severe hypoglycemia, for which overview results were not statistically significant, Vardi and colleagues showed a reduction of 27% (OR 0.73; 95% CI 0.61; 0.87) and for nocturnal hypoglycemia, the results were in the same direction, with a significant reduction favouring insulin analogue (OR 0.70; 95% CI 0.63; 0.79) [30]. The results of Singh and colleagues’ review [31] are very similar to ours. Nocturnal hypoglycemia was reduced in insulin analogues in 35% (OR 0.65; 95% CI 0.55;0.77) [31].

Regarding clinical trials, it is worth noting that first pilot studies are usually misunderstood. Considering dose titration phases, the proportion of patients experiencing hypoglycemia episodes during the titration period was higher for insulin analogues than for NPH in the first trials [26,38,39,70,71]. Titration could bring several biases to the interpretation of the results, considering the use of equal doses for NPH and analogues. Thus, a substantial number of hypos in the analogues group was noted. In conclusion, neither hypoglycemia episodes nor A1C in the titration period should be included in the results, according to Food and Drug Administration recommendations: “The primary efficacy parameter should be assessed substantially after the end of the titration period (e.g., 3 months) to better reflect the steady-state effect of the dose regimens studied” [69].

Two other major challenges when analyzing RCTs in T1D are the different ways of measuring hypoglycemia and the short follow-up of studies. Regarding the definition of hypoglycemia, this varies across the studies (<2.0 mmol / L, <2.8 mmol / L, <3.1 mmol / L, <4.0 mmol / L), contributing 14% to the heterogeneity of general hypoglycemia in our analysis. Recommendations from the American Diabetes Association Workgroup on Hypoglycemia [72] and the International Society for Pediatric and Adolescent Diabetes [73], which stratify and define hypoglycemia appropriately, have been published. However, many of the studies, published later than these recommendations, do not use them.

Regarding the duration of the studies, it is important to criticize studies with less than 12 weeks of follow-up. This is insufficient time to capture changes in a chronic disease, mainly related to A1C, which is known to take three months (or 12 weeks) to present changes caused by interventions or treatments [69,73,74].

Some authors and regulatory agencies around the world point to the lack of efficacy of insulin analogues for three reasons: presentation of results based on a surrogate endpoint; absence or small magnitude of difference in A1C; and high heterogeneity in meta-analytical analysis. These points are controversial, as we highlight in the following paragraphs.

Regarding account A1C as a surrogate outcome, researchers in evidence-based medicine, among them the creators of the GRADE instrument, have proposed quality criteria to evaluate studies with surrogate outcomes. These authors [75] have stated that surrogate outcomes should be considered relevant if they can establish a direct relationship between the surrogate outcome and the clinical outcome it is intended to replace. The evidence of significance for A1C occurs through the largest clinical trial in T1D and its extension, DCCT (1) and EDIC [76]. The results of these studies established the relationship between a 1% decrease in A1C and at least a 43% decrease in microvascular complications. Such reduction of 1% is internationally accepted as clinically significant [69].

Regarding the small magnitude of the effect of long-acting insulin analogues in A1C, it is important to remember that its primary role in the algorithm for treating T1D is to improve glucose control and decrease the risk of hypoglycemia. Therefore, since A1C is an average of the glycaemia of the last three months, we can see the fact that these insulins decrease the occurrence of hypoglycemia (lower limit of the mean) as positive, providing maintenance or even little improvement of A1C [74], as occurs in this overview.

Heterogeneity was high and significant, being greater than 90% in all outcomes considered in our overview. The variables that most explained the heterogeneity were bolus insulin (26%) and definition of hypo (14%) for general hypoglycemia, cross-over design (23%) and intervention brand (13%) for nocturnal hypoglycemia, and Jadad score (16%) for A1C. Two other factors have influenced the inconsistency of the results, but their impact was not clear: i) the fact that the rate of episodes per person-time was calculated for most of the studies, and the absence of standard deviation estimates for A1C in some meta-analysis. High heterogeneity was common among the systematic reviews analyzed in this overview [10,30,31]. It is worth noting that a portion of the heterogeneity could not be explained by the variables studied in this overview.

Some limitations of our study should be acknowledged. First, we did not include insulin degludec in our overview. Although it is one the most recent long-acting insulins on the market, we did not identify any study comparing it to NPH. Second, we excluded some conference abstracts due to the low methodological quality of the reports, often with insufficient data for analysis. Third, when we searched for new RCTs published after the last systematic review included in our overview, we only searched Pubmed, i.e. we did not carry out a full systematic review of RCTs.

To date, some indirect comparison analyses have been performed [10,11,77], but none of them included all the therapeutic options available in terms of basal insulins. Thus, as a recommendation for future research, we suggest performing indirect comparison analysis or network meta-analysis including all basal profile insulins, such as NPH, glargine, detemir, degludec [78], lispro protamine suspension [79], peglispro [80], glargine 300 U/ml [81], pre-mixed [82], and biosimilars insulins [83]. This latter may represent hope for population access to better insulins at an affordable cost for public health systems in developing countries [84–87].

## Supporting information

S1 Checklist PRISMA checklist. (DOCX)

S1 Fig Risk of bias on included randomized clinical trials. (TIF)

S1 Table Search strategies. (DOCX)

S2 Table Characteristics of included systematic reviews. (DOCX)

S3 Table Characteristics of excluded systematic reviews. (DOCX)

S4 Table The AMSTAR score of included systematic reviews. (DOCX)
