# Supplementary figures and images for: Long-acting insulin analogues for type 1 diabetes: An overview of systematic reviews and meta-analysis of randomized controlled trials
Source: PLoS One. 2018 Apr 12;13(4):e0194801. doi: 10.1371/journal.pone.0194801 (PMC5896894; doi:10.1371/journal.pone.0194801)

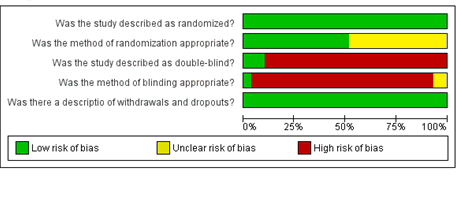

Supplement: S1 Fig — (TIF) [file pone.0194801.s002.tif]
