# Supplementary material for: Long-acting insulin analogues for type 1 diabetes: An overview of systematic reviews and meta-analysis of randomized controlled trials
Source: PLoS One. 2018 Apr 12;13(4):e0194801. doi: 10.1371/journal.pone.0194801 (PMC5896894; doi:10.1371/journal.pone.0194801)
Supplement: S1 Table — (DOCX) [file pone.0194801.s003.docx]

| **DATABASE** | **SEARCH STRATEGY** |
| --- | --- |
| EMBASE | 'insulin dependent diabetes mellitus'/mj OR 'insulin dependent diabetes mellitus' OR 'type 1 diabetes':ab,ti OR 'diabetes mellitus type 1':ab,ti OR 'diabetes type 1':ab,ti OR t1d:ab,ti AND ('long-acting insulin analogue':ab,ti OR 'long acting insulin analogue':ab,ti OR 'long-acting insulin analog*':ab,ti OR 'long-acting analog* insulin':ab,ti OR glargine:ab,ti OR lantus:ab,ti OR 'hoe 901':ab,ti OR detemir:ab,ti OR levemir:ab,ti OR nn304:ab,ti OR degludec:ab,ti OR 'insulin degludec':ab,ti) AND ([cochrane review]/lim OR [systematic review]/lim OR [meta analysis]/lim) AND ([english]/lim OR [portuguese]/lim OR [spanish]/lim) |
| Cochrane Library | type 1 diabetes (Title, Abstract, Keywords) AND long acting insulin analogue (Search All Text) |
