# Supplementary material for: Long-acting insulin analogues for type 1 diabetes: An overview of systematic reviews and meta-analysis of randomized controlled trials
Source: PLoS One. 2018 Apr 12;13(4):e0194801. doi: 10.1371/journal.pone.0194801 (PMC5896894; doi:10.1371/journal.pone.0194801)
Supplement: S2 Table — (DOCX) [file pone.0194801.s004.docx]

| **STUDY** | **DESIGN** | **INTERVENTION VS COMPARATOR** |
| --- | --- | --- |
| Wang et al, 2003 [26] | Systematic review without meta-analysis | Glargine vs human insulin |
| Warren et al, 2004 [27] | Health Technology Assessment | Glargine vs human insulin |
| Mullins et al, 2007 [28] | Systematic review with meta-analysis (Negative Binomial Meta-Regression Analysis) | Glargine vs human insulin |
| Tran et al, 2007 [29] | Health Technology Assessment | Long-acting insulin analogues vs human insulin |
| Vardi et al, 2008 [30] | Systematic review with meta-analysis | Long-acting insulin analogues vs human insulin |
| Singh et al, 2009 [31] | Systematic review with meta-analysis | Insulin analogues vs human insulin |
| Sanches et al, 2011 [11] | Mixed treatment comparison | Long-acting insulin analogues vs human insulin |
| Szypowska et al, 2011 [32] | Systematic review with meta-analysis | Detemir vs human insulin |
| Frier et al, 2013 [33] | Systematic review without meta-analysis | Detemir vs human insulin |
| Souza et al, 2014 [34] | Systematic review without meta-analysis | Glargine vs human insulin |
| Tricco et al. 2014 [10] | Network meta-analysis | Long-acting insulin analogues vs human insulin |
