# Supplementary material for: Long-acting insulin analogues for type 1 diabetes: An overview of systematic reviews and meta-analysis of randomized controlled trials
Source: PLoS One. 2018 Apr 12;13(4):e0194801. doi: 10.1371/journal.pone.0194801 (PMC5896894; doi:10.1371/journal.pone.0194801)
Supplement: S3 Table — (DOCX) [file pone.0194801.s005.docx]

| **STUDY** | **JADAD** | **POPULATION** | **TREATMENT PERIOD (weeks)** | **INTERVENTION x COMPARATOR**  **(daily frequency)** | **SAMPLE SIZE** | **BOLUS** | **OUTCOMES** |
| --- | --- | --- | --- | --- | --- | --- | --- |
| Raskin et al, 2000 [38] | 3 | Adults | 16 | Glargine (od) x human insulin (od or bid) | 310 versus 309 | Lispro | General hypoglycemia  Severe hypoglycemia  Nocturnal hypoglycemia  A1C |
| Ratner et al, 2000 [39] | 2 | Adults | 28 | Glargine (od) x human insulin (od or bid) | 264 versus 270 | Regular | General hypoglycemia  Severe hypoglycemia  Nocturnal hypoglycemia  A1C |
| Hermansen et al, 2001 [40] | 2 | Adults | 12 | Detemir (od) x human insulin (od) | 57 versus 56 | Regular | General hypoglycemia  Severe hypoglycemia  Nocturnal hypoglycemia |
| Schober et al, 2002 [41] | 3 | Children | 28 | Glargine (od) x human insulin (od or bid) | 175 versus 175 | Regular | A1C |
| Rossetti et al, 2003 [42] | 2 | Adults | 12 | Glargine (bid) x human insulin (4) | 17 versus 17 | Lispro | General hypoglycemia  Nocturnal hypoglycemia  A1C |
| Vague et al, 2003 [43] | 3 | Adults | 26 | Detemir (bid) x human insulin (bid) | 301 versus 146 | Aspart | General hypoglycemia  Severe hypoglycemia  Nocturnal hypoglycemia  A1C |
| Murphy et al, 2003 [44] | 2 | Adolescents | 16 | Glargine (od) x human insulin (od) | 25 versus 25 | Lispro vs Regular | General hypoglycemia  Nocturnal hypoglycemia  A1C |
| Porcellatti et al, 2004 [45] | 3 | Adults | 52 | Glargine (od) x human insulin (4) | 61 versus 60 | Lispro | General hypoglycemia  Nocturnal hypoglycemia  A1C |
| Hermansen et al, 2004 [46] | 2 | Adults | 12 | Detemir (bid) xhuman insulin (bid) | 298 versus 297 | Aspart vs Regular | General hypoglycemia  Nocturnal hypoglycemia  A1C |
| Home et al, 2004 [47,48] | 3 | Adults | 12 | Detemir (bid) x human insulin (bid) | 276 versus 132 | Aspart | General hypoglycemia  Severe hypoglycemia  Nocturnal hypoglycemia  A1C |
| Russel-Jones et al, 2004 [49] | 3 | Adults |  | Detemir (od) x human insulin (od) | 491 versus 256 | Regular | General hypoglycemia  Severe hypoglycemia  Nocturnal hypoglycemia  A1C |
| Standl et al, 2004 [50] | 2 | Adul |  | Detemir (bid) x human insulin (bid) | 154 versus 135 | Regular | General hypoglycemia  Severe hypoglycemia  Nocturnal hypoglycemia  A1C |
| Home et al, 2005 [51] | 3 | Adults | 28 | Glargine (od) x human insulin (od or bid) | 292 versus 293 | Regular | A1C |
| Fulcher et al, 2005 [52] | 3 | Adults | 30 | Glargine (od) x human insulin (od) | 62 versus 63 | Lispro | General hypoglycemia  Severe hypoglycemia  Nocturnal hypoglycemia  A1C |
| Pieber et al, 2005 [53] | 2 | Adults | 12 | Detemir (bid) x human insulin (bid) | 271 versus 129 | Aspart | General hypoglycemia  Severe hypoglycemia  Nocturnal hypoglycemia  A1C |
| NN304-1476 [54] | 2 | Adults | 44 | Detemir (od or bid) x human insulin (od or bid) | 178 versus 95 | Aspart | General hypoglycemia  Nocturnal hypoglycemia  A1C |
| Ashwell et al, 2006 [55] | 3 | Adults | 16 | Glargine (od) x human insulin (od or bid) | 51 versus 51 | Lispro vs Regular | General hypoglycemia  Nocturnal hypoglycemia  A1C |
| Kolendorf et al, 2006 [56] | 2 | Adults | 10 | Detemir (bid) x human insulin (bid) | 125 versus 128 | Aspart | General hypoglycemia  Severe hypoglycemia  Nocturnal hypoglycemia  A1C |
| Robertson et al, 2007 [57] | 3 | Children and adolescents | 26 | Detemir (od or bid) x human insulin (od or bid) | 232 versus 115 | Aspart | General hypoglycemia  Severe hypoglycemia  Nocturnal hypoglycemia  A1C |
| Chatterjee et al, 2007 [58] | 3 | Adults | 16 | Glargine (od) x human insulin (bid) | 57 versus 57 | Aspart | General hypoglycemia  Severe hypoglycemia  Nocturnal hypoglycemia  A1C |
| Mianovska et al, 2007 [59] | 2 | Children | 24 | Glargine (od) x human insulin | 14 versus 14 | Lispro or Regular | A1C |
| Chase et al, 2008 [60] | 2 | Adolescents | 24 | Glargine (od) x human insulin or Lente (bid) | 85 versus 90 | Lispro | General hypoglycemia  Severe hypoglycemia  A1C |
| Bartley et al, 2008 [61] | 3 | Adults | 104 | Detemir (od) x human insulin (od) | 331 versus 164 | Aspart | General hypoglycemia  Severe hypoglycemia  Nocturnal hypoglycemia  A1C |
| Hassan et al, 2008 [62] | 3 | Children and adolescents |  | Glargine (bid) x human insulin (bid) | 19 versus 17 | Lispro or Aspart | General hypoglycemia  A1C |
| Bolli et al, 2009 [63] | 2 | Adults | 24 | Glargine (od) x human insulin (bid or +) | 85 versus 90 | Lispro | General hypoglycemia  Severe hypoglycemia  A1C |
