# Supplementary material for: Long-acting insulin analogues for type 1 diabetes: An overview of systematic reviews and meta-analysis of randomized controlled trials
Source: PLoS One. 2018 Apr 12;13(4):e0194801. doi: 10.1371/journal.pone.0194801 (PMC5896894; doi:10.1371/journal.pone.0194801)
Supplement: S4 Table — (DOCX) [file pone.0194801.s006.docx]

| Systematic reviews | AMSTAR item | | | | | | | | | | | |
| --- | --- | --- | --- | --- | --- | --- | --- | --- | --- | --- | --- | --- |
|  | 1 | 2 | 3 | 4 | 5 | 6 | 7 | 8 | 9 | 10 | 11 | # of YES |
| Wang, 2003 [26] | N | N | Y | Y | N | Y | N | N | N | N | N | 3 |
| Warren, 2004 [27] | N | N | Y | Y | Y | Y | Y | Y | Y | N | Y | 8 |
| Mullins, 2007 [28] | N | N | Y | N | N | Y | N | N | N/A | N | Y | 3 |
| Tran, 2007 [29] | Y | Y | Y | Y | Y | Y | Y | Y | Y | N | Y | 10 |
| Vardi, 2008 [30] | Y | Y | Y | Y | Y | Y | Y | Y | Y | Y | Y | 11 |
| Singh, 2009 [31] | Y | Y | Y | Y | N | Y | Y | Y | Y | Y | Y | 10 |
| Sanches, 2011 [11] | N | Y | Y | Y | N | Y | Y | N | Y | N | Y | 7 |
| Szypowska, 2011 [32] | N | Y | Y | Y | Y | Y | Y | N | Y | N | Y | 8 |
| Frier, 2013 [33] | N | N | N | N | N | Y | Y | N | N | N | Y | 3 |
| Souza, 2014 [34] | Y | Y | Y | N | N | Y | Y | Y | NR | N | Y | 7 |
| Tricco, 2014 [10] | Y | Y | Y | Y | N | Y | Y | Y | Y | Y | Y | 10 |

Keys: N, no; NR, not reported; Y, yes (systematic review fulfilling the criteria); # of Yes, number of yes; AMSTAR item: 1. Was an 'a priori' design provided? 2. Was there duplicate study selection and data extraction? 3. Was a comprehensive literature search performed? 4. Was the status of publication (i.e. grey literature) used as an inclusion criterion? 5. Was a list of studies (included and excluded) provided? 6. Were the characteristics of the included studies provided? 7. Was the scientific quality of the included studies assessed and documented? 8. Was the scientific quality of the included studies used appropriately in formulating conclusions? 9. Were the methods used to combine the findings of studies appropriate? 10. Was the likelihood of publication bias assessed? 11. Was the conflict of interest included?
